# Patient Perception of Depressive Symptoms in Rheumatic Diseases: A Cross-sectional Survey

**DOI:** 10.1097/RHU.0000000000001564

**Published:** 2020-09-14

**Authors:** Francesca Ingegnoli, Tommaso Schioppo, Tania Ubiali, Silvia Ostuzzi, Valentina Bollati, Massimiliano Buoli, Roberto Caporali

**Affiliations:** From the ∗ Division of Clinical Rheumatology, ASST Pini; † Department of Clinical Sciences & Community Health, Research Center for Adult and Pediatric Rheumatic Diseases, Research Center for Environmental Health, Università degli Studi di Milano; ‡ ALOMAR Lombard Association for Rheumatic Diseases; § EPIGET—Epidemiology, Epigenetics and Toxicology Lab, Università degli Studi di Milano; ∥ Department of Neurosciences and Mental Health, Fondazione IRCCS Ca’Granda Ospedale Maggiore Policlinico; ¶ Department of Pathophysiology and Transplantation, Università degli Studi di Milano, Milano, Italy.

**Keywords:** depression, mood disorder, patient perspective, PHQ-9, research, rheumatic diseases

## Abstract

**Objectives:**

The presented study aimed to explore the presence and the self-identification of depressive symptoms among patients with rheumatic musculoskeletal diseases (RMDs) through the use of the Patient Health Questionnaire (PHQ-9).

**Methods:**

Between June and October 2019, patients from the regional association for people with RMDs in Lombardy, Italy (ALOMAR), were invited to participate in a cross-sectional online survey. Participants completed PHQ-9 along with a survey about their perception of depressive symptoms. Patients were stratified according to PHQ-9 score as follows: not depressed (<4), subclinical or mild depression (5–9), moderate depression (10–14), moderately severe depression (10–14), and severe depression (20–27). Descriptive statistics and analyses of variance were used to explore data.

**Results:**

Of the 192 RMD patients who completed PHQ-9, 35 (18.2%) were not depressed, 68 (35.4%) had subclinical or mild depression, 42 (21.9%) had moderate depression, 30 (15.6%) had moderately severe depression, and 17 (8.9%) had severe depression. Contrary to the above findings, only 16 respondents (8.3%) reported that they experienced depressive symptoms, and only 7 of the 16 were being followed by a psychiatrist. Respondents with higher PHQ-9 scores tended to have concomitant fibromyalgia, to be younger, and to be overweight.

**Conclusions:**

The current results indicate the overall burden of depressive symptoms in RMD patients. While clinical depression (PHQ-9 >10) was detected in 41.2% of respondents, only 8.3% reported that they experience depressive symptoms. Routine screening of RMD patients for depression is therefore critical.

While major depression is one of the most prevalent and disabling disorders worldwide, it is often undiagnosed and therefore undertreated.^1^ Depressive symptoms play an important role in rheumatic musculoskeletal diseases (RMDs), representing one of the most common medical comorbidities and the most frequent psychiatric conditions associated with RMD.^2–6^ Rheumatic musculoskeletal diseases are a heterogeneous group of disorders with a high rate of morbidity and mortality.^7–10^ Thus, the concomitant presence of depressive symptoms among RMD patients implies a considerable economic and social burden.^7–11^

Depressive symptoms are associated with numerous deleterious outcomes, including increased mortality, work disability, disease activity, treatment noncompliance, physical dysfunction, pain, and fatigue.^3,12,13^ Moreover, depression impacts negatively on patient global assessment, thus diminishing clinical remission. While it is increasingly apparent that depressive symptoms are an intrinsic component of RMDs, they are still poorly recognized and managed in these patients.^2,3,14–16^

Although the co-occurrence of RMDs and depressive symptoms has been widely described, a common pathogenic pathway is currently not well understood. Recent evidence suggests shared pathways on the basis of circadian oscillations of inflammatory mediators, such as tumor necrosis factor α and interleukin 6 (IL-6), in patients with RMDs and depressive symptoms.^17^ Moreover, it has been demonstrated that IL-6 plays a crucial role in inducing and worsening mood disturbances by both increasing inflammation and directly affecting nociceptive neurons and the hypothalamic-pituitary-adrenal axis. These findings are critical to the optimization of treatment choices in patients with inflammatory arthritis.

A considerable proportion of patients with RMD respond poorly to therapy. However, the reason for this is currently not well understood. It is widely accepted that depressive symptoms may reduce the odds of full treatment efficacy and therefore diminish the probability of improvement over time.^12^

Despite a growing interest in the association between depressive symptoms and RMD, the self-identification of depressive symptoms among RMD patients is currently not known. Therefore, the current cross-sectional online study explored the perception of depressive symptoms among Italian patients with RMDs in contrast to the existence of depressive symptoms as measured by the Patient Health Questionnaire 9 (PHQ-9).

## METHODS

### Survey Design and Administration

The current nonprofit cross-sectional study was conducted online to screen for the presence and the self-identification of depressive symptoms in patients with RMDs. The ethical committee of the University of Milan granted approval for all study procedures (20.05.19-18/19). Between June and October 2019, the online questionnaire was disseminated to the patient association ALOMAR using a mailing list, website, and social networks (http://www.alomar.it/). Data protection was ensured by the information technology (IT) service of the Università degli Studi di Milano (Italy).

Participants were not remunerated and gave voluntary consent to complete the survey. All patient responses remained anonymous. The survey included questions about basic patient demographics and background information. Patients were asked to self-report disease characteristics and comorbidities. The results of the study were summarized by means and percentages and reported according to the Guidance for Reporting Involvement of Patients and the Public checklist.^18,19^

### Patient Health Questionnaire 9

The PHQ-9, one of the most widely used depression scales in clinical practice,^20^ was administered to patients with RMDs.^6,21,22^ A recent manuscript confirmed the validity and reliability of PHQ-9 to assess depressive symptoms in patients affected by RMDs.^23^

The PHQ-9^16^ rates frequency of symptoms over the past 2 weeks on a 0- to 3-point Likert-type scale (from “not at all” to “nearly every day”), with total scores ranging from 0 to 27.^20^ The items on PHQ-9 are derived from the *Diagnostic and Statistical Manual of Mental Disorders, Fourth Edition* classification system and rate (1) anhedonia, (2) depressed mood, (3) trouble sleeping, (4) feeling tired, (5) change in appetite, (6) feelings of guilt or worthlessness, (7) trouble concentrating, (8) feeling slowed down or restless, and (9) suicidal thoughts. Patients were stratified according to PHQ-9 cutoff scores as follows: not depressed (<4), subclinical or mild depression (5–9), moderate depression (10–14), moderately severe depression (15–19), and severe depression (20–27).

### Statistical Analysis

Descriptive statistics were used to summarize patient demographics, clinical data, and PHQ-9 results. Patients were grouped according to self-reported diagnosis as follows: group 1 (inflammatory arthritis), patients with rheumatoid arthritis (RA), psoriatic arthritis, and ankylosing spondylitis; group 2 (connective tissue diseases/vasculitis), patients with systemic lupus erythematous, undifferentiated or mixed connective tissue disease, systemic sclerosis, inflammatory myositis, Sjögren syndrome, and vasculitis; and group 3 (miscellaneous RMDs), all patients with RMDs other than those listed in groups 1 and 2 (osteoarthritis, polymyalgia rheumatica, fibromyalgia, and crystal arthropathies).

A 1-way analysis of variance was performed to compare PHQ-9 scores among the 3 groups. In addition, a multivariate linear regression analysis was conducted to explore the association between PHQ-9 score (dependent variable) and disease duration, age, age at diagnosis, sex, presence of obesity/overweight, and presence of fibromyalgia (independent variables). All analyses were performed using R software, version 3.5.2, with package Rcmdr (version 2.5–1).

## RESULTS

A total of 192 RMD patients (124 with inflammatory arthritis, 49 with connective tissue diseases/vasculitis, and 19 with miscellaneous RMDs) responded to PHQ-9. One hundred seventy respondents were women (88.5%) with a median age of 50 years. All results were obtained from questionnaire responses; no medical records were reviewed. Gastroesophageal reflux disease was the most frequently reported comorbidity and was present in 37 of 192 patients (19.2%) (Table 1).

**TABLE 1:** Characteristics of Patients With RMDs

	Inflammatory Arthritis**^a^**	Connective Tissue Disease**^b^**	Miscellaneous**^c^**	Total
No. patients	124	49	19	192
Sex, male/female, n	16/108	46/3	16/3	78/114
Age, median (IQR), y	49 (37.8–56)	48 (38–60)	52 (50–60)	50 (38–57.3)
Disease duration, median (IQR), y	9 (3–15)	6 (1–13)	6 (0.5–9.5)	7 (3–14)
Age at diagnosis, median (IQR), y	37.5 (22.8–47)	38 (27–48)	48 (41–51)	38.5 (26.8–48)
Secondary fibromyalgia, n (%)	25 (20.2%)	6 (12.2%)	—	31 (16.1%)
Concomitant OA, n (%)	9 (7.3%)	2 (4%)	—	11 (5.7%)
Secondary sicca syndrome, n (%)	6 (4.8%)	5 (10.2%)	—	11 (5.7%)
Comorbidities, n (%)				
Arterial hypertension	22 (17.7%)	5 (10.2%)	4 (21%)	31 (16.1%)
Diabetes	5 (4%)	1 (2%)	1 (5.3%)	7 (3.6%)
CV disease	8 (6.5%)	1 (2%)	3 (15.8%)	12 (6.2%)
Overweight/obesity	16 (12.9%)	3 (6.1%)	4 (21%)	23 (12%)
Depressive symptoms	8 (6.5%)	3 (6.1%)	5 (26.3%)	16 (8.3%)
Anxiety	12 (9.7%)	4 (8.1%)	6 (31.6%)	22 (11.5%)
Gastritis	10 (8%)	3 (6.1%)	4 (21%)	17 (8.9%)
GERD	20 (16.1%)	9 (18.4%)	8 (42%)	37 (19.3%)
Other GI disease	20 (16.1%)	2 (4%)	5 (26.3%)	27 (14%)
Thyroiditis	16 (12.9%)	11 (22.5%)	4 (21%)	31 (16.1%)
Ocular diseases	13 (10.4%)	5 (10.2%)	3 (15.8%)	21 (%)

^a^Rheumatoid arthritis, psoriatic arthritis, ankylosing spondylitis.

^b^Connective tissue disease and vasculitis.

^c^Osteoarthritis, gout, chondrocalcinosis, polymyalgia, primary fibromyalgia.

CV, cardiovascular; GERD, gastroesophageal reflux disease; GI, gastrointestinal; IQR, interquartile range; OA, osteoarthritis.

According to PHQ-9 score, of the 192 respondents, 35 (18.2%) were not depressed. Depression was subclinical or mild in 68 (35.4%), moderate in 42 (21.9%), moderately severe in 30 (15.6%), and severe in 17 patients (8.9%). Among the participants with depression, only 16 (8.3%) perceived themselves to be depressed, and 7 of the 16 were followed by a psychiatrist.

**FIGURE 1:**
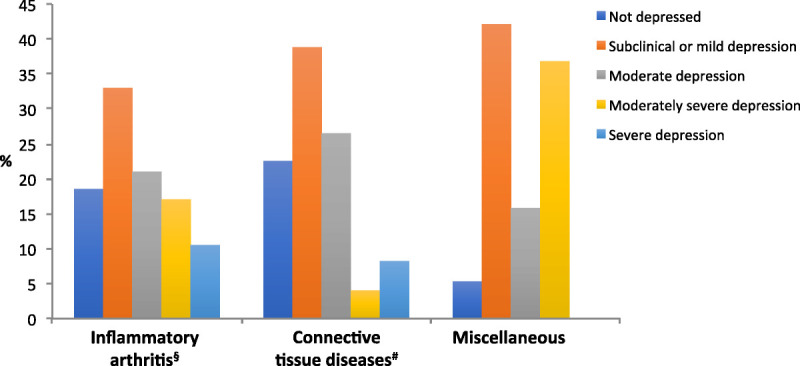
Depressive symptom severity according to PHQ-9 among groups of patients with inflammatory arthritis, connective tissue diseases, and miscellaneous RMDs. ^§^Rheumatoid arthritis, psoriatic arthritis, ankylosing spondylitis. ^#^Connective tissue disease and vasculitis. *Osteoarthritis, gout, chondrocalcinosis, polymyalgia, primary fibromyalgia.

There were no statistically significant differences in PHQ-9 scores between the 3 RMD groups (*p* = 0.2733) (Fig. 1). In group 1 with inflammatory arthritis (n = 124), 23 participants (18.5%) were not depressed. Depression was subclinical or mild in 41 (33%), moderate in 26 (21%), moderately severe in 21 (17%), and severe in 13 (10.5%). Among participants in group 1, only 8 (6.5%) self-identified as depressed, and only 3 of the 8 were followed by a psychiatrist.

In group 2 with connective tissue diseases and vasculitis (n = 49), 11 (22.5%) were not depressed. Depression was subclinical or mild in 19 (38.8%), moderate in 13 (26.5%), moderately severe in 2 (4%), and severe in 4 (8.2%). Among participants in group 2, only 3 (6%) self-identified as depressed, and only 1 of the 6 was followed by a psychiatrist.

In group 3 with miscellaneous RMDs (n = 19), 1 participant (5.3%) was not depressed, whereas 8 (42.1%) had subclinical or mild depression, 3 (15.8%) had moderate depression, and 7 (36.8%) had moderately severe depression. Among participants in group 3, 5 (26.3%) self-identified as depressed, and 3 of the 5 were being followed by a psychiatrist.

**FIGURE 2:**
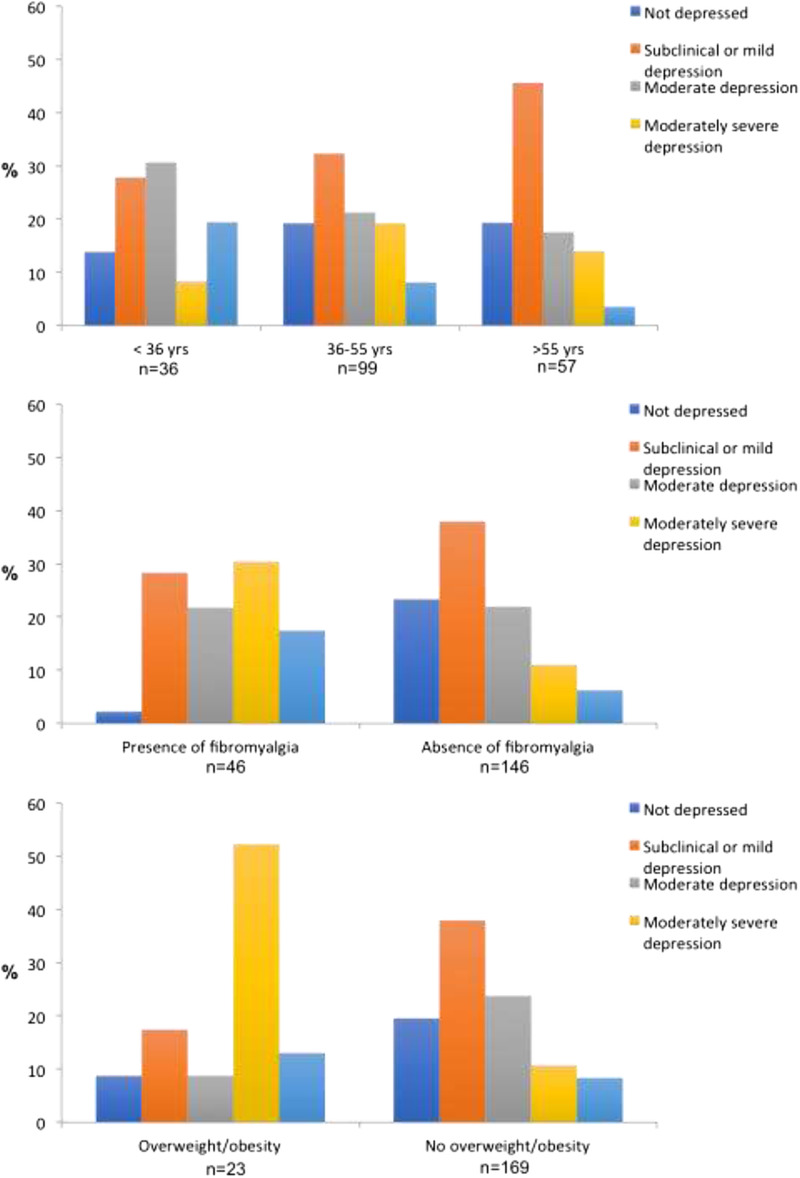
Depressive symptom severity according to PHQ-9 scores among patients with RMDs stratified by age, fibromyalgia, and weight disorder.

Finally, factors that have known comorbidities with depressive symptoms, such as fibromyalgia, being overweight and age were analyzed (Table 2 and Fig. 2). Of the 192 respondents, 46 had fibromyalgia, and 23 were overweight/obese. A multivariate linear regression analysis found a significant association between depressive symptom severity and age (estimate: −0.059; *p* = 0.046), fibromyalgia (estimate: 4.364; *p* < 0.001), and weight (estimate: 4.380; *p* < 0.001).

**TABLE 2:** Multivariate Linear Regression Analysis for the Association Between Depressive Symptom Severity as Measured by PHQ-9 and Known Risk Factors

Independent Variable Description	Estimate	SE	*p* value
Disease duration	0.029	0.040	0.469
Age at visit	−0.059	0.029	0.046
Fibromylagia	4.364	0.953	<0.001
Overweight/obesity	4.380	4.380	<0.001
Sex	−2.302	1.262	0.069

## DISCUSSION

The current study highlights the burden of depressive symptoms among patients with RMDs, along with a lack of patient insight into this problem. Of note, 46.4% of the respondents scored more than 10 on PHQ-9, indicating clinical depression. However, only 8.3% of these patients self-identified as depressed, and even fewer (4%) were regularly followed up by a psychiatrist.

The percentage of RMD patients with depressive symptoms in the current study was higher than that previously reported.^24–26^ The prevalence for depression among individuals with RA has been reported as ranging from 15% to 39%,^26^ whereas the percentage of patients with systemic lupus erythematosus who experience depression is between 24% and 39%.^25^ Furthermore, the overall risk of depression among RA patients has been reported to be 2 to 3 times higher than that in the general population.^24^

In agreement with previous studies, the current study found significant associations between depressive symptoms and age, fibromyalgia, and being overweight. Interestingly, prior studies have postulated that the association between depression and being overweight/obese is bidirectional.^6,27^ One explanation for this finding is based on the inflammatory theory of depression in which proinflammatory cytokines, such as IL-6 and tumor necrosis factor α, have been reportedly observed in patients with depression, and concurrently, inflammation is correlated with metabolic abnormalities (e.g., leptin).^28,29^

Despite the high prevalence of depressive symptoms, few participants self-identified as depressed. One explanation for this finding is that depressive symptoms, including pain and fatigue, are also associated with RMDs,^30^ and therefore, patients who experience these symptoms ascribe them to their disease and not to depression. In addition, patients may be apprehensive of the stigma associated with psychiatric disorders and be reluctant to speak about depressive symptoms with doctors and relatives.^31^ Alternatively, patients may not have the opportunity to discuss psychiatric issues with their rheumatologists or general practitioners.^31^ In fact, prior studies have reported that rheumatologists seldom inquire about their patients’ mental health.^31,32^ However, considering the negative consequences of neglecting depressive symptoms, such as declining physical disability, poor quality of life, and increased suicidal risk, rheumatologists should monitor their patients’ mental health.^33^ Furthermore, RMD patients with depressive symptoms tend to be less compliant with medical treatment than patients without depression.^34^ These findings further support the importance of early recognition and treatment of depression in patients with RMDs.

The high prevalence of depression in individuals affected by RMDs suggests the possibility of a common biological pathway. Pain and physical disability associated with RMDs have been shown to increase inflammation and cause abnormalities in circadian rhythm, which in turn worsen physical functioning and can cause persistence of depressive symptoms.^35^ This deleterious loop can be disrupted by early diagnosis and appropriate treatment of mood disorders, leading to better mental health in patients affected by RMDs.^35^

Taken together, the findings from the current and prior studies underline the importance of the establishment of guidelines for the management of depressive symptoms in patients affected by RMDs. Specifically, a collaboration between rheumatologists and psychiatrists is vital for the management of the most severe patients. The presented study provides important data on the mental health of patients living with RMDs. There were many strengths to the study design. For example, the participants were assured anonymity, thus avoiding the potential confounding factor of fear about social stigmas. In addition, participants were able to respond to questionnaires online, avoiding confounding factors associated with the type of clinical setting where they were being treated (e.g., university clinics vs. general hospitals). However, limitations of the study include the cross-sectional design, the small sample size, and the potential biased selection of patients who are more confident with IT and therefore more prone to respond to online surveys.

Future research should better clarify shared underlying biological mechanisms of depression and RMDs and explore whether antidepressants can modify inflammatory parameters and, conversely, if inflammatory modulators, such as immunosuppressant therapies, can improve mood symptoms.

In conclusion, considering the current and previously reported findings, the importance of screening psychiatric comorbidities, particularly mood disorders, in patients with RMDs is critical. Treatment of comorbid psychiatric conditions is likely to improve the global course of RMDs.

## KEY POINTS

- Depressive symptoms are common among rheumatic patients, but are often not identified.
- Depressive symptoms in rheumatic patients are related to age, fibromyalgia, and weight disorders.
